# Establishment and characterization of 24 breast cancer cell lines and 3 breast cancer organoids reveals molecular heterogeneity and drug response variability in malignant pleural effusion-derived models

**DOI:** 10.1186/s13058-025-02032-7

**Published:** 2025-05-01

**Authors:** Soon-Chan Kim, Ga-Hye Kim, Jae-Hyeon Park, Kyung-Hun Lee, Jiwon Koh, Tae-Yong Kim, Dae-Won Lee, Yu-Jin Kim, Seongyeong Kim, Song-Yi Park, Ahrum Min, Young-Kyoung Shin, Seock-Ah Im, Ja-Lok Ku

**Affiliations:** 1 Cancer Research Institute, Seoul National University, Seoul, 03080 Republic of Korea; 2 Department of Biomedical Sciences, Seoul National University College of Medicine, Seoul, 03080 Republic of Korea; 3 Medical Research Center, Ischemic/Hypoxic Disease Institute, Seoul National University College of Medicine, Seoul, 03080 Republic of Korea; 4 Department of Internal Medicine, Seoul National University Hospital, Seoul National University College of Medicine, Seoul, 03080 Republic of Korea; 5 Department of Pathology, Seoul National University Hospital, Seoul, 03080 Republic of Korea; 6 Department of Internal Medicine, Seoul Metropolitan Government Seoul National University Boramae Medical Center, Seoul, 07061 Republic of Korea; 7 Division of Data Science, College of Information and Communication Technology, The University of Suwon, Hwaseong, 18323 Republic of Korea

**Keywords:** Intratumoral Heterogeneity, Breast Cancer, Malignant Pleural Effusion, Drug Screening, Next Generation Sequencing

## Abstract

Intratumoral heterogeneity of breast cancer cells causes undesired drug resistance and predispose to disease recurrence. We investigate the molecular heterogeneity of breast cancer cells derived from malignant pleural effusions (MPE) to understand variations in drug resistance and cellular evolution. MPE provides a unique environment, with cells experiencing significant microenvironmental changes that promote intratumoral heterogeneity and therapeutic resistance. By establishing 24 cell lines and 3 organoids from MPE samples of breast cancer patients, we performed extensive genomic, transcriptomic, and drug sensitivity analyses. Our findings reveal substantial genetic and transcriptomic diversity among MPE-derived cell lines, highlighting dynamic alterations in driver mutations and signaling pathways that correlate with variable drug responses. Notably, temporal and spatial heterogeneity within patient-derived samples emphasized the adaptability of breast cancer cells in MPE, as different subclones displayed distinct drug sensitivities. This work underscores the critical role of molecular profiling in understanding treatment resistance in breast cancer, presenting MPE-derived cell lines as valuable preclinical models for exploring personalized therapies in aggressive cancer phenotypes.

**Supplementary Information:**

The online version contains supplementary material available at 10.1186/s13058-025-02032-7.

## Introduction

Breast cancer is the most frequently diagnosed malignancy worldwide [1], and the leading cause of cancer-related death in females [2]. The rapid evolution of breast cancer results in highly heterogeneous clonal composition, contributing to therapeutic resistance and dismal prognosis [3]. Both spatial and temporal heterogeneity contribute to significant variances in tumor characteristics such as proliferation, metastasis, and treatment response [4]. However, most prior studies have focused on spatial variations in tumor properties [5, 6], leaving the longitudinal dynamics of molecular profiles in breast cancer largely unexplored [7].

Malignant pleural effusion (MPE) is observed in 5–11% of breast cancer patients, being the second most common after lung cancer. It is considered as a dismal prognostic factor, and the therapeutic response in MPE-complicated breast cancer is generally poor [8, 9]. Previous studies have shown that tumor cells in MPE develop resistance to anoikis, adapt to hypoxic stress, and exhibit epithelial-mesenchymal transition (EMT), contributing to multidrug resistance and metastatic potential [10–12]. Moreover, comprehensive mutational profiling of MPE supernatants has demonstrated their potential as non-invasive surrogates for predicting therapeutic resistance. These malignant cells bring about unpredictable drug resistance and potentially spread to the pleural membrane and adjacent organs [13]. These findings underscore the need to further investigate drug resistance mechanisms in MPE-derived tumor cells to inform personalized treatment strategies.

MPE has been a primary source for establishing breast cancer cell lines, including widely used cell lines such as MDA-MB-134, MDA-MB-175, and MDA-MB-231 [14]. Tumor cells that invade the pleural cavity retain the majority of genetic alterations present in primary breast tumors, demonstrating their clinical value in diagnosis [15, 16]. In this study, we established 24 MPE-derived in vitro models of breast cancer, including 13 cell lines derived from heavily treated breast cancer patients, which were subjected to genomic, transcriptomic, and in vitro drug sensitivity analyses. Our approaches aimed to investigate the molecular heterogeneity of breast cancer cells evading in the pleural cavity and to correlated with variable drug responses.

## Materials and Methods

### Establishment of breast cancer cell lines and organoids

The research protocol was reviewed and approved by the institutional review board of the Seoul National University Hospital (IRB No. 1102–098–357). The study was performed in accordance with the Declaration of Helsinki. Written informed consent was obtained from all patients enrolled in this study. All samples were from patients in Seoul National University Hospital (Seoul, Republic of Korea), and all participants were of Korean (East Asian) ethnicity.

Twenty cell lines were established from MPE and one cell line was established from malignant ascites. Three cell lines and organoids were gained from the patient-derived xenograft (PDX), which was established from the body fluids of cancer patients; this was due to the scant cellularity of the body fluids, and we needed to expand the cellularity within the PDX to get a sufficient number of cancer cells.

For cell lines from MPE and ascites, suspended cells were gathered by spinning down. Gathered cell pellet were seeded into T-25 or T-75 flasks. Cancer cells were initially cultured in Opti-MEM I (Thermo Fisher Scientific, MA, USA) with 5% fetal bovine serum (FBS). A confined area trypsinization or scraping method was used to attain achieve pure tumor cells when stromal cells like mesothelial cells or fibroblasts grew in the initial culture. After primary culture, established cell lines were sustained in RPMI 1640 (Thermo Fisher Scientific) with 10% fetal bovine serum and 1% (v/v) penicillin and streptomycin (10,000U/mL). Incubated flasks in humidified incubators at 37℃ in an atmosphere of 5% CO2 and 95% air.

For cell lines and organoids established from PDX tissues, PDX tumor tissue was cut finely with scissors for approximately 5 min. The enzyme solution consisting of Collagenase II (1.5 mg/mL), Hyaluronidase (20 µg/mL) and Ly27632 (10 µM) was added to the chopped tissue and incubated for 4 h at 37 ℃ while spinning. FCS was added for neutralization and the mixture was filtered through a 100 µM cell strainer (SPL, #93,100) to remove large chunks and impurities that were not cut well. Cells were spun down at 1,000 rpm for 3 min. For cell line cultures, same method previously introduced in cell lines culture was applied. For organoid cultures, the cell pellet was resuspended with the appropriate amount of basement membrane extracts (BME) gel (Gibco, A14132-02), and the mixture was plated in droplets of 50–100 µL each. The mixture was left for 10 min to allow the BME gel to solidify, and then the HBEC medium (Basal culture medium with 50% Wnt conditioned medium, 20% R-Spondin conditioned medium, 10% Noggin conditioned medium, 1 × B27 (Gibco, 17,504–044), 1.25 mM n-Acetyl cysteine (Sigma, A7250), 5 mM Nicotinamide, 5 nM Neuregulin (Peprotech, 100–03), 500 nM A83-01, 500 nM SB202190 (Sigma, S7067), 5 mM Ly27632, 5 ng/mL human EGF (Peprotech, AF-100–15), 20 ng/mL human FGF-10 (Peprotech, 100–26), 5 ng/mL human FGF-7 (Peprotech, 100–19) and 50 µg/mL Primocin (Thermo, ant-pm-1)) was added and incubated at 37 ℃. Established cell lines and organoids were deposited to Korean Cell Line Bank (KCLB, Seoul, Republic of Korea) and will be distributed upon request.

### Maintenance of breast cancer organoids

The culture medium was aspirated, and the BME gel was mechanically dissociated through repeated pipetting. The mixture of organoids and gel was centrifuged at 1,000 rpm for 3 min, and the medium was suctioned. Approximately 5 mL of Triple Express (Invitrogen) was added, and the mixture was incubated at 37 ℃ for approximately 10 min. After 5 min, the size of the organoids was checked and the gel was removed every minute. To neutralize, FCS and medium were added, and loose cells were spun down at 1,500 rpm for 3 min. After mixing the pellet with the appropriate amount of gel, the mixture was plated in droplets of 50–100 µL each. The mixture was left for 10 min to allow the BME gel to solidify, and then the HBEC medium was added. The medium was typically changed every week.

### Growth properties and morphology in vitro

To determine the doubling time for each tumor population, 5 × 10^3^/mL to 2 × 10^4^/mL viable cells from each cell line were seeded into 12–24 identical wells of a 96 well-plate. Cell viability was assessed daily for 5–12 days. Starting from the initial seeding, every 24 h, 10 µL of EZ-Cytox solution (Daeil Lab, Seoul, Republic of Korea) was added to each well containing breast cancer cells, in triplicate. After a 2 h incubation at 37℃, the optical density of the EZ-Cytox-treated cells was measured using the Multiskan™ GO Microplate Spectrophotometer (Thermo Fisher Scientific, MA). Growth rate values were calculated using GraphPad Prism 5 (GraphPad Software, CA, USA). To observe the morphology of each cell line, cells were cultured in T-75 flasks and photographed daily using phase-contrast microscopy.

### Genomic DNA extraction and DNA fingerprinting analysis

Genomic DNA (gDNA) was extracted using the QIAamp DNA Mini kit (Qiagen). gDNA extracted from each breast cancer cell line and organoid was amplified using an AmpFlSTR identifier Polymerase Chain Reaction (PCR) Amplification Kit (Applied Biosystems, CA, USA). A single cycle of PCR amplified 15 short tandem repeat markers (CSF1PO, D2S1338, D3S1358, D5S818, D7S820, D8S1179, D13S317, D16S539, D18S51, D19S433, D21S11, FGA, TH01, TPOX and VWA) and an amelogenin gender-determining marker containing highly polymorphic microsatellite markers. Amplified PCR products were analyzed using an ABI 3500XL Genetic analyzer (Applied Biosystems).

### Genomic DNA Mycoplasma detection test

gDNA extracted from each breast cancer cell line and organoid was amplified using an TaKaRa PCR Mycoplasma Detection Set (TAKARA BIO INC., Shiga, Japan). This kit allows detection of several different species of Mycoplasma (M. fermentans, M. hyorhinis, M. arginini, M. orale, M. salivarium, M. hominis, M. pulmonis, M. arthritidis, M. neurolyticum, M. hyopneumoniae, M. capricolum) and one species of Ureaplasma (U. urealyticum). The method involves amplifying the spacer regions in the rRNA operon specifically the region between the 16S and 23S genes, which were designed based on the DNA encoding of the 16S and 23S rRNAs.

### Drug sensitivity analysis using two-dimension (2D) cell lines culture models

To measure the drug sensitivity, 5 × 10^4^/mL to 2 × 10^5^/mL viable cells from each cell line were seeded into a well of 96-well white plate (SPL, #30,196) in triplicate. One day later, all cell lines and organoids were treated with appropriate drug concentrations After 72 h of incubation at 37 °C, 10 µL of Cell-titer Glo solution was added to each well. Following a 20-min incubation at 37 °C, the luminescence was measured using the Multiskan™ Ascent Microplate Luminometer (Thermo Fisher Scientific). This procedure was repeated in duplicate. Drug sensitivity was assessed using the Area Under the Curve (AUC) method.

### Drug sensitivity analysis using three-dimension (3D) organoids models

Organoids were placed around the rim of the well of 96 well white plates (SPL, #30,196) in a 1:1 mixture of HBEC medium and RGF basement membrane matrix (Gibco, A14132-02). Plates were incubated at 37 ℃ with 5% CO2 for 15 min to solidify the gel. After solidification, 20 µL of pre-warmed HBEC medium was added to each well. After 96 h, 20 µL of serially diluted drug solutions were added to each well, with the control wells receiving a mixture of HBEC medium and drug solvent solution. Following a 20-min incubation at 37 °C, the luminescence was measured again using the Multiskan™ Ascent Microplate Luminometer. This procedure was repeated in duplicate. Drug sensitivities were presented as AUC with six dilution points, using drugs mostly selected from the NIH breast cancer medical supplies list (https://www.cancer.gov/about-cancer/treatment/drugs/breast) along with a few recently identified anti-cancer compounds.

### Confocal analysis of immunofluorescence staining

Cells were seeded on chambered cover glass (Thermo Fisher Scientific) to achieve desirable confluence. Seventy-two hours after seeding, cells were fixed and permeabilized using BD Cytofix/Cytoperm™ (BD science, CA, USA). After washing with BD washing solution, cells were blocked with DPBS containing 2% FBS (GE Healthcare Life Sciences, Buckinghamshire, UK) for an hour. Following a cold DPBS wash, cells were incubated with HER2 (Santa Cruz Biotechnology, CA, USA) and E-cadherin antibodies (Abcam, Cambridge, UK) diluted to 0.05% in PBS-T for 1.5 h at room temperature. Cells were then washed with 0.05% PBS-T and incubated with Alexa 488 and Alexa 594 secondary antibodies (Thermo Fisher Scientific) diluted in 0.05% PBS-T for an hour at room temperature. After staining with 1 × DAPI (Sigma-Aldrich, MO, USA) diluted in distilled water for 30 min at room temperature, cells were washed three times with DPBS and imaged using a confocal microscope.

### Histopathologic analysis

Tumor tissues were fixed in 10% neutral buffered formalin, embedded in paraffin, and sectioned at 4 µm thickness. For organoids, the BME dome was mechanically scraped with a pipette tip. Cold PBS (10 mL) was added to collect the dissociated BME domes, which were transferred to a 15 mL conical tube. After centrifuging for 15 s at 100 rpm, the supernatant was aspirated. This procedure was repeated until the BME gel was visibly removed, taking care not to damage the original structure of the organoids. Collected organoids were embedded in 2% agarose gel (INTRON Biotechnology, Seongnam, Republic of Korea). The solidified agarose gel was fixed in 10% formalin for 30 min at room temperature and sectioned at 4 µm thickness. The sections were then subjected to H&E staining.

### Whole Exome Sequencing of cell lines

Total DNA was isolated from the cell line and organoid pellets using QIAamp DNA Mini Kit (Qiagen, Hilden, Germany) according to the manufacturer’s protocol. For cell lines, SureSelect sequencing libraries were prepared following the manufacturer’s instructions (Agilent SureSelectXT Human All Exon V4) using The Bravo automated liquid handler. The captured targets were sequenced using Illumina Novaseq 6000 system (Illumina, San Diego, CA, USA) with the pair-end 100 bp read option. For organoids, whole-exome capture was performed with the SureSelect Human All Exon V5 Kit (Agilent Technologies, Tokyo, Japan). Captured targets were sequenced using HiSeq 2500 (Illumina) with the pair-end 100 bp read option for organoid samples.

The sequence data from cell lines and organoids were processed through an in-house pipeline. Briefly, paired-end sequences were aligned to the human reference genome (UCSC assembly hg19—original GRCh37 from NCBI, 2009) using the mapping program BWA (version 0.7.12) [17], and generated a mapping result file in BAM format using BWA-MEM. PCR duplicates were removed using MarkDuplicates.jar included in Picard tools (v. 1.130, https://broadinstitute.github.io/picard/). The Genome Analysis Toolkit (GATK, v.3.4.0) [18] was used to performed base quality score recalibration (BQSR) and local realignment around indels. Haplotype Caller of GATK (GATKv3.4.0) was used for variant genotyping of each sample based on the BAM file previously generated (SNP and short indel candidates are detected). Those variants are annotated by SnpEff v4.1 g, to vcf file format, annotating with dbSNP for the version of 142 and SNPs from the 1000 genome project. Then, SnpEff was applied to annotate additional databases, including ESP6500, ClinVar, dbNSFP 2.9.

### Analysis of RNA sequencing

Total RNA was isolated from cell lysate using TRIzol (Qiagen) and Qiagen RNeasy Kit (Qiagen). Library was prepared with TruSeq Stranded mRNA LT Sample Prep Kit in accordance with TruSeq Stranded mRNA Sample Preparation Guide, Part #15,031,047 Rev. E. Paired-end sequencing reads (101 bp) from cDNA libraries were generated with an Illumina NovaSeq6000 instrument, and sequence quality was verified using FastQC v.0.11.7 (https://www.bioinformatics.babraham.ac.uk/projects/fastqc/). For data preprocessing, low quality bases and adapter sequences were trimmed using Trimmomatic v 0.38 [19]. The trimmed reads were aligned to the human genome (UCSC hg19) using HISAT v2.1.0, a splice-aware aligner [20]. Then, transcripts including novel splice variants were assembled with StringTie v1.3.4 d [21]. The abundance of these transcripts in each sample was calculated as read counts or TPM (Transcript per Million mapped reads) values.

### Statistical analysis

Statistical analysis was performed using R program version 3.3.1 (R Foundation for Statistical Computing, Vienna, Austria) with packages including maftools, PerformanceAnalytics, survminer, survival, iplot, gplot, and lattice. Fisher’s exact test was used to analyze GO analysis of various genes. A multivariate analysis of variance (MANOVA) model was applied to the drug response data matrix, considering factors such as the mutational status and the three different transcriptional subtypes. Approximate F value, p-value and Pillai’s trace score were obtained for each of the factors/drug pairs. A value of p < 0.05 was considered statistically significant. For hierarchical cluster analysis on a set of dissimilarities, each object was assigned to its own cluster, which an algorithm proceeds through iteratively. Two of the most similar clusters are joined at each stage until there is a single cluster. Distances between clusters are recomputed at each stage by the Lance–Williams dissimilarity update formula according to the particular clustering method used. Clustering methods include Ward's minimum variance method, complete linkage method, k-means method, and single linkage method.

## Results

### Breast cancer in vitro models retained heterogeneous morphological features of the primary tissue

We have established 24 breast tumor lines in which twenty from the MPE, one from the ascites, and three cell lines were accrued from PDX models of patients who had scant numbers of malignant cells in the body fluids. For a better readability, multiple cell lines established from a same patient were categorized with patient numbers as follows: Multiple set 1 (SNU-3223, SNU-3224, SNU-3230), Multiple set 2 (SNU-3380, SNU-3393), Multiple set 3 (SNU-3698 A, SNU-3698B, SNU-3698 C, SNU-3705, SNU-3716, SNU-3730) and Multiple set 4 (SNU-5188, SNU-5226B). Detailed clinicopathological characteristics of 15 enrolled patients in this study were summarized in Table 1 and Table S1. DNA fingerprinting analysis showed a heterogeneous distribution of 15 tetranucleotide repeat loci and an amelogenin gender (Table S2). All models were confirmed to be free of mycoplasma contamination (Fig. S1).

**Table 1:** Clinicopathological characteristics of 15 enrolled breast tumor patients

No	SNU Name	Multiple set	Race/Ethnicity	Origin	Growth patterns	Doubling time (hours)	Morphology	Sex/Age	Tumor type	Clinical diagnosis	Histology subtype	IHC subtype	ER (+, %)	PR (+, %)	HER2 IHC	Ki-67(%)
1	**SNU-2480**		Korean (East Asian)	Pleural effusion	Adherent/Floating	17.6	Oval	F/37	Metastatic	Relapsed	IDC	TNBC	Negative	Negative	Negative	60
2	**SNU-2532 A**		Korean (East Asian)	Pleural effusion	Floating	9.9	Round	F/32	Metastatic	Relapsed	IDC	TNBC	Negative	Negative	1 +	-
3	**SNU-2924**		Korean (East Asian)	Pleural effusion	Adherent/Floating	6	Round/Polygonal	F/46	Primary	Relapsed	IDC	TNBC	Negative	Negative	Negative	50
4	**SNU-3129**		Korean (East Asian)	Pleural effusion	Adherent	2.3	Fibroblast-like	F/53	Metastatic	Relapsed	IDC	TNBC	Negative	Negative	Negative	5
5	**SNU-3171**		Korean (East Asian)	Ascites	Adherent	8.7	Fibroblast-like/Polygonal	F/56	Metastatic	Relapsed	IDC	HR +	Positive, 60%	Negative	2 + (FISH-)	-
6	**SNU-3196**		Korean (East Asian)	Pleural effusion	Adherent/Floating	-	Polygonal	F/70	Metastatic	Relapsed	IDC	TNBC	Focal weak	-	Negative	-
7	**SNU-3223**	**Multiple set 1**	Korean (East Asian)	Pleural effusion	Adherent	6	Polygonal	F/41	Metastatic	Relapsed	IDC	HER2 +	Negative	Negative	3 +	60
8	**SNU-3224**				Adherent	7.6	Polygonal									
9	**SNU-3230**				Adherent/Floating	18.9	Round/Polygonal									
10	**SNU-3351**		Korean (East Asian)	Pleural effusion	Adherent	19	Polygonal	F/65	Metastatic	Relapsed	ILC	HR +	Positive, 90%	Positive, 50%	2 + (-)	-
11	**SNU-3380**	**Multiple set 2**		Pleural effusion	Adherent	8	Polygonal	F/63	Metastatic	Relapsed	IDC	HER2 +	Negative	Negative	3 +	-
12	**SNU-3393**				Adherent	76.5	Polygonal									
13	**SNU-3698 A**	**Multiple set 3**	Korean (East Asian)	Pleural effusion	Adherent/Floating	8	Round	F/36	Metastatic	De Novo stage IV	IDC	HER2 +	Negative	Negative	3 +	-
14	**SNU-3698B**				Adherent/Floating	23.4	Round									
15	**SNU-3698 C**				Floating	5.1	Round									
16	**SNU-3705**				Adherent/Floating	5.8	Round									
17	**SNU-3716**				Adherent/Floating	10.9	Round									
18	**SNU-3730**				Adherent/Floating	6.7	Round									
19	**SNU-4842**		Korean (East Asian)	PDX tissue	Floating	10.1	Round	F/44	Primary	Relapsed	IDC	HER2 +	Negative	Negative	3 +	30
20	**SNU-4842-TO**															
21	**SNU-4856**		Korean (East Asian)	PDX tissue	Adherent	-	Round	F/44	Metastatic	Relapsed	IDC	HR +	Negative	Positive, 70%	Negative	10
22	**SNU-4856-TO**															
23	**SNU-5126**		Korean (East Asian)	PDX tissue	Adherent	4.7	Polygonal	F/53	Metastatic	Relapsed	IDC	HR +	Positive, 95%	Negative	Negative	-
24	**SNU-5126-TO**															
25	**SNU-5188**	**Multiple set 4**	Korean (East Asian)	Pleural effusion	Adherent	60.5	Fibroblast-like/Round	F/62	Metastatic	De Novo stage IV	IDC	HR +	Positive, 80%	Positive, 5%	Negative	7
26	**SNU-5226B**				Adherent/Floating	12	Fibroblast-like/Polygonal									
27	**SNU-5884B**		Korean (East Asian)	Pleural effusion	Adherent	4.3	Polygonal	F/48	Metastatic	De Novo stage IV	IDC	TNBC	Negative	Negative	Negative	-

*IDC* Invasive Ductal Carcinoma, *ILC* Invasive Lobular Carcinoma, *TNBC* Triple Negative Breast Cancer (ER-PR-HER2-), *HR +*  Hormone Receptor positive (ER + and/or PR +).

**Fig. 1:**
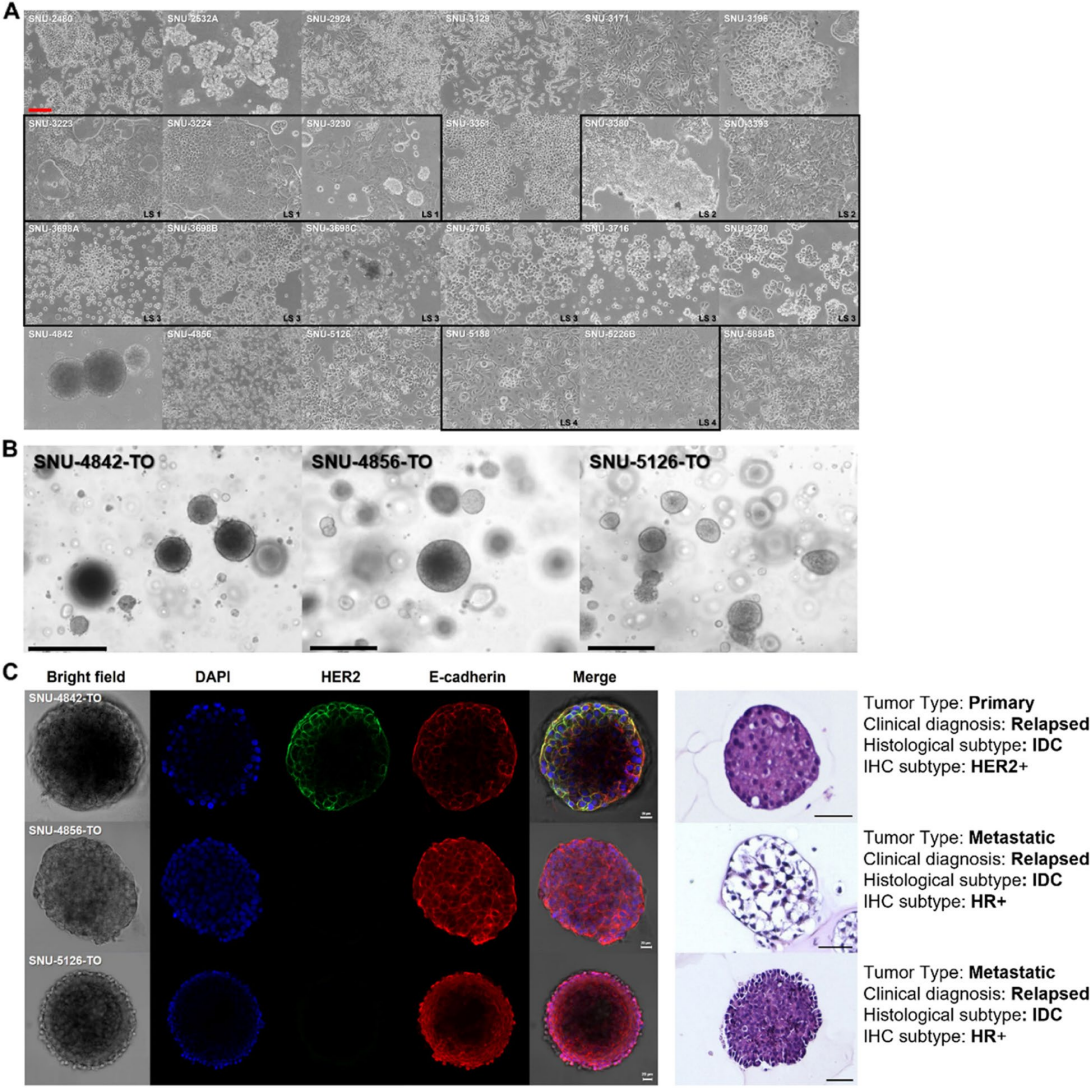
The morphologies of MPE-derived human breast cancer In Vitro models exhibited heterogeneous patterns. **A** Majority of cell lines grow as adherent form, suggesting cells floating in the pleural cavity maintained the capability to adhere. Various growth patterns include polygonal (*n* = 8), fibroblast-like (*n* = 1), round (*n* = 9), oval (*n* = 1) and mixed (*n* = 5) form. The names of cell lines are indicated upper-left corner. Multiply collected samples from the same patient are indicated with black squares. Scale bar = 200 μm. **B** Bright field images of three PDX-derived organoids displayed mostly spheroidal and asymmetric morphologies. Scale bar = 100 μm. **C** Immunocytochemistry of PDX-derived organoids and hematoxylin–eosin (H&E) staining of paraffin sections demonstrated organoids maintained the characteristics of the original breast tumor. Scale bar = 20 μm

The morphologies of the established cell lines were mainly classified into four types: polygonal, oval, round and fibroblast-like (Fig. 1A, Table 1). Cell lines mostly exhibited adherent patterns despite of their pleural effusion origin, which implied that breast cancer cells in the pleural cavity retained the characteristics of anchorage-dependent growth. Interestingly, different growth patterns were observed in cell lines derived from the same patients. For instance, in Multiple set 3, SNU-3716 cell lines mostly grew as both adherent and floating aggregates whereas SNU-3730 cell line exhibited predominantly adherent patterns in similar numbers of passages. Although in vitro culture conditions might shape the cellular population differently, these cell lines partially demonstrated that the growth patterns may vary within the MPE microenvironment (Fig. 1A).

Tumor cells harvested from three patients (SNU-4842, SNU-4856 and SNU-5126) were initially used to establish PDX models subjected to further organoid culture. They showed mostly spheroidal and asymmetric morphologies and matched cell lines grew as monolayers of substrate-adherent cells displaying polygonal and spindle shapes (Fig. 1B). H&E staining of paraffin sections from the tumor organoids (TO) indicated that SNU-4856-TO was loosely aggregated compared to two other compact organoids. Immunocytochemistry (ICC) demonstrated that SNU-4842-TO had clear expressions of HER2, in line with the histopathological diagnosis of the patient (Fig. 1C, Table 1). As shown in Fig. 1C, organoids derived from HER2-positive patients, such as SNU-4842, exhibited clear HER2 expression, whereas organoids derived from HER2-negative patients, including SNU-4856 and SNU-5126, did not show HER2 staining, reflecting the immunohistochemical profiles of the original tumors. These suggests that organoids established from MPE via PDX model still retained the characteristics of MPE.

### Multiple sampling and modeling of breast tumor cells within pleural cavity revealed intratumoral heterogeneity population

We performed whole exome sequencing (WES) to estimate the variant allele frequencies (VAFs) of driver mutations. The mutational profiling of the breast tumors was focused on 700 driver genes documented in Cancer Gene Census [22] and ClinVar database [23] in order to validate the pathogenicity of selected mutations. All marked mutations in the Fig. S2 are either pathogenic or conflicting interpretations of pathogenicity in the ClinVar database. (Fig. S2 A, Table S3 A, B). The mutational landscape indicated that *TP53* and *APOB* exhibited the highest mutation frequencies at 56% and 52%, respectively. Multiple sample sets overall showed similar mutational pattern.

**Fig. 2:**
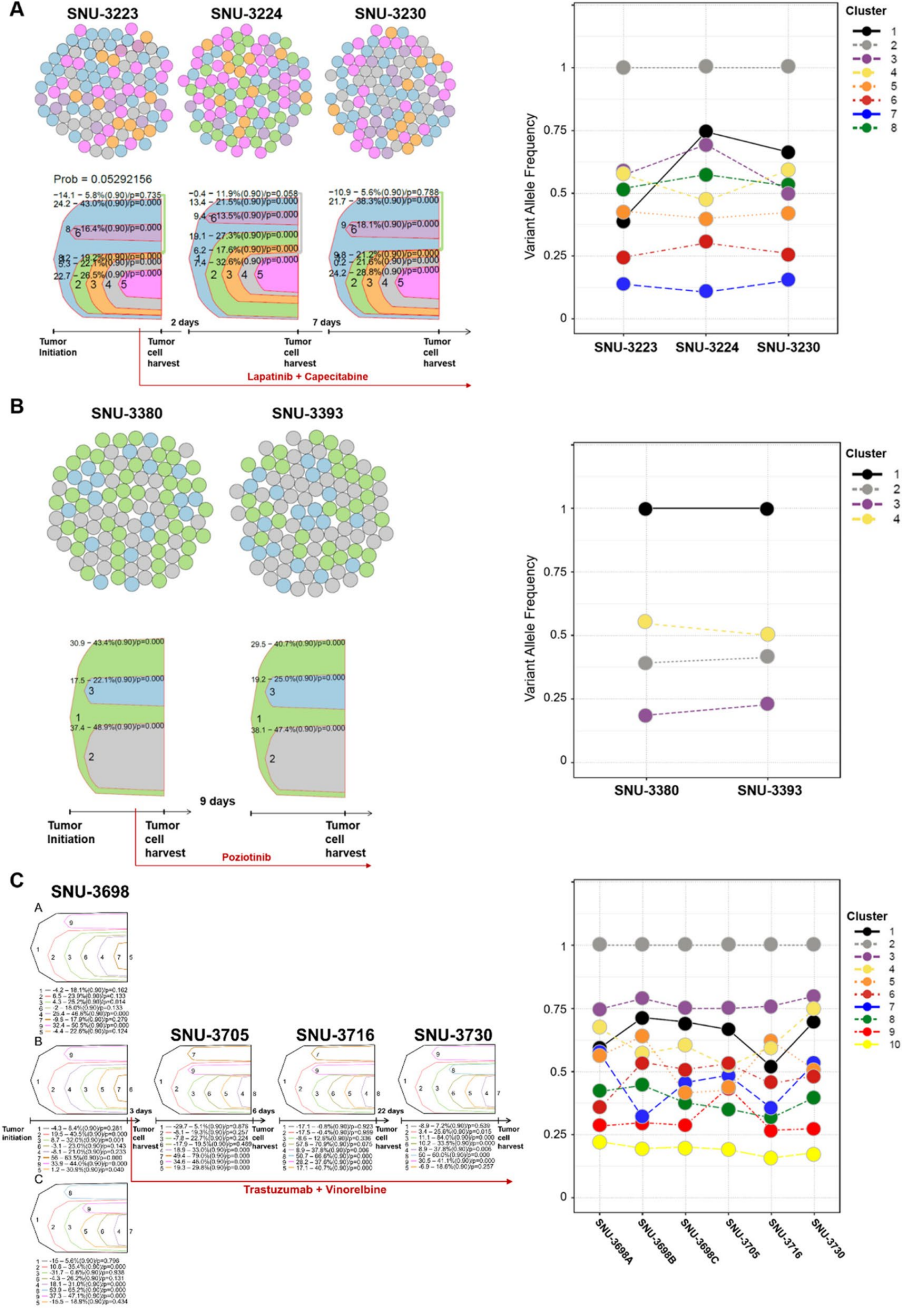
Intratumoral heterogeneity within malignant pleural effusions of breast cancer patients. **A**-**C** Clonal estimation of the multiple set 1–3. Calculated subclones and their proportions among a tumor population within the MPE are indicated with colored circles and fish plots using representative colors. The number of colored circles per each harvesting point is set to 100. The time intervals for collecting tumor cells and applied antitumor drugs are indicated below the fish plot. The VAFs of mutational clusters per multiple collected samples are indicated with a broken line graphs with representative colors. The clone numbers corresponding to the line graphs in Fig. 2 are provided on the right side of each graph, and the associated gene mutations with significantly changed VAFs are detailed in Table S4 A–4 C. The subclones of multiple set 4 could not be estimated with identical parameters and omitted from the clonal analysis. See also Table S3 A-3 C and S4 A-4 C

Cell lines originating from the same patient mostly shared overall landscapes of the mutational features. To trace the heterogeneity of breast cancer cells evading into the pleural cavity, we assessed the continuously altering VAFs. For instance, the tumor cells from the Multiple set 1 (SNU-3223, SNU-3224, and SNU-3230) were serially harvested within a week. The patient received lapatinib and capecitabine before collecting cancer cells from MPE when patients were progressed after lapatinib and capecitabine (Fig. 2A). Although the estimated number of subclones within the pleural cavity did not vary, the proportion of subclones was different with elevated VAFs of *IRF6*, *SPTA1, FLT3,* and *ATRX* genes (Table S4 A)*.* On the other hands, the Multiple set 2 (SNU-3380 and SNU-3393) were collected after progression from pan-HER inhibitor posiotinib showed that both the composition and proportion of the subclones within the pleural cavity remained largely unchanged (Fig. 2B, Table S4B). The Multiple set 3 represented spatiotemporal heterogeneity as three samples (SNU-3698 A, SNU-3698B, and SNU-3698 C) were harvested on the same day (after progression from T-DM1) yet cultured separately, and the rest (SNU-3705, SNU-3716, and SNU-3730) were collected during 3rd line trastuzumab with vinorelbine at a short interval of days. The spatially heterogeneous samples (SNU-3698 A, SNU-3698B, and SNU-3698 C) exhibited two different clonal compositions which were maintained throughout subsequent harvest (Fig. 2C, Fig. S2B, Fig. S2 C and Table S4 C). For instance, SNU-3716 which was collected 9 days after the drainage of SNU-3698 series showed similar composition comparable to SNU-3698 C. In contrast, SNU-3730, which was harvested 31 days after the collection of SNU-3698, exhibited analogous constitution of SNU-3698 A and SNU-3698B.

### Transcriptomic analysis revealed patient-specific expressional patterns

**Fig. 3:**
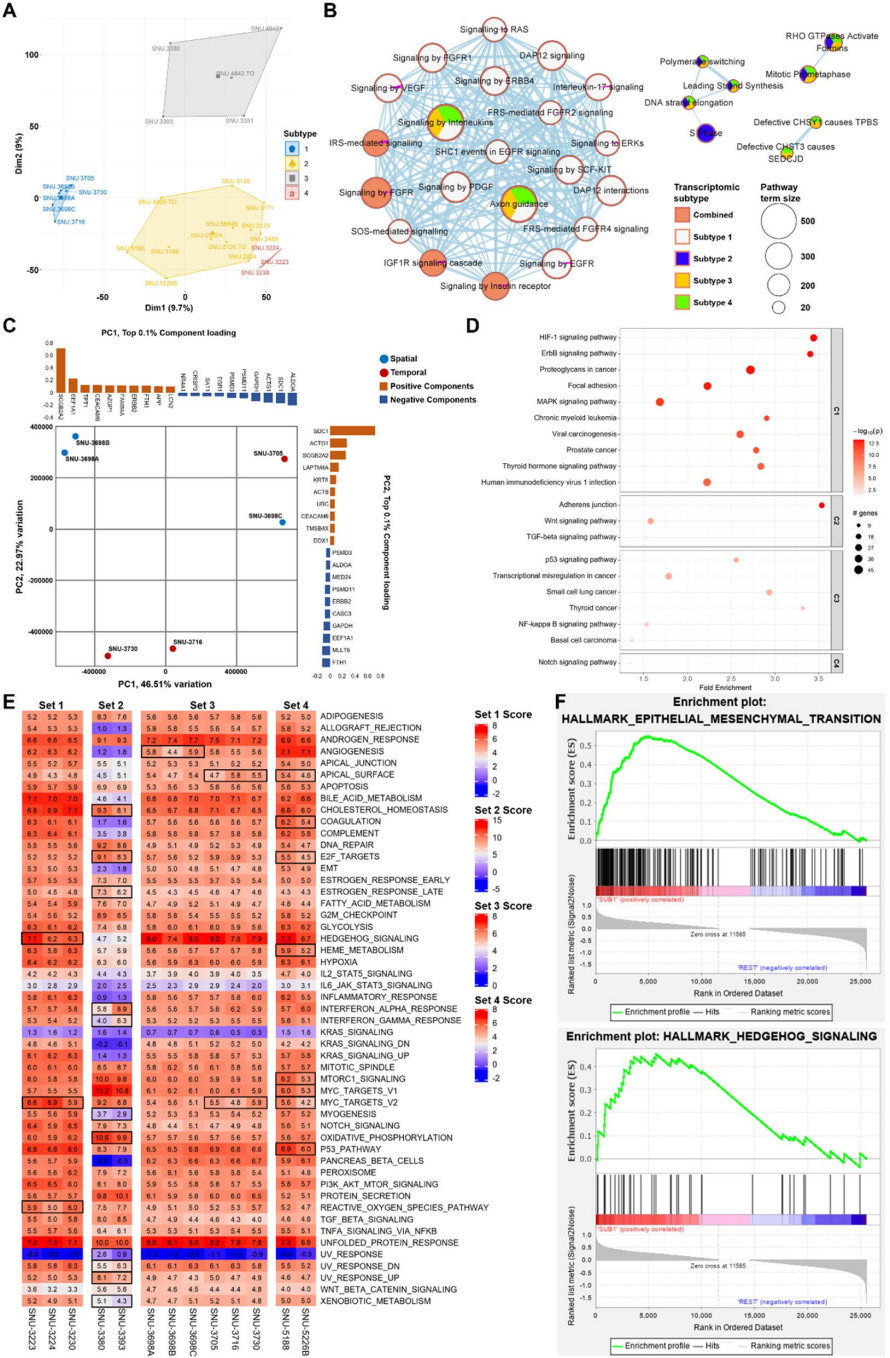
Multiple sets of MPE-derived breast tumor cell lines revealed patient-specific transcriptomic alteration. **A** Principle component analysis (PCA) showed high level of inter-patient heterogeneity. A total of 4 transcriptomic subtypes are identified with PCA. Each subtype is indicated with connected lines and representative colors. **B** See also Table S5. Multivariate pathway enrichment analysis identifies gene sets that are over-represented across transcriptomic subtypes. Four transcriptomic subtypes identified from PCA are used to estimate pathway aberrations, each indicated by a representative color. Pathways associated with all four subtypes are labeled as “Combined.” The size of each circle corresponds to the pathway term size, representing the number of genes in each pathway. Only pathways with statistically significant enrichment (adjusted *p* < 0.05) are shown. The filled colors within each circle represent the transcriptomic subtypes contributing to the enrichment of each pathway. **C** See also Table S6. PCA of Multiple set 3 validates spatiotemporal heterogeneity of MPE-derived breast tumor cell. The principle component 1 (PC1) separates spatially heterogeneous samples with 46.51% of total variation, whereas the principle component 2 (PC2) divides temporally heterogeneous samples with 22.97% of total variation (*n* = 27,681). Top 0.1% component loadings of PC1 and PC2 were indicated on the top and right side of the PCA plot respectively. **D** See also Table S7. Pathway enrichment analysis of loading components that account for temporal heterogeneity. The log-transformed p-values are indicated with gradient colors. A value of p <.05 is considered statistically significant. The size of the circle is proportional to the sample size. The fold enrichment is indicated on the x-axis, and the names of enriched pathways are indicated on the y-axis. **E** Table S8. Normalized enrichment scores (NES) for each Multiple set using single sample gene set enrichment analysis (ssGSEA) display overall temporal transcriptomic changes of MPE samples. Pathways with standard deviation > 0.5 of NES were highlighted with black squares. **F** EMT and hedgehog signaling pathways are distinctively expressed in the set 2. The statistical settings for GSEA analysis is as follows (Number of permutations = 1000, Permutation type = phenotype, Chip platform = MSigDB.v.7.4.chip, Enrichment statistic = weighted, Max size: exclude larger sets = 500, Min size: exclude smaller sets = 15)

We further investigated if the changing microenvironment of MPE was associated with transcriptomic alteration. We first analyzed the overall mRNA expression pattern using principal component analysis (PCA), which revealed a high level of intertumoral heterogeneity (Fig. 3A). Two transcriptomic subtypes 1 and 4 entirely represented Multiple sets 3 and 1, displaying patient-specific expression patterns. Although transcriptomic Clusters 2 and 3 displayed a high degree of dispersion in PCA, we conducted sub-clustering within these groups. While smaller sub-clusters were identified, the limited number of samples in each precluded statistically robust pathway enrichment analysis. These limitations underscore the need for cautious interpretation of inter-patient differences in gene expression patterns. We then estimated which signaling pathways were involved with each subtype from the PCA (Fig. 3B). Overall, signaling by interleukins and axon guidance pathways were largely modified in subtypes 1, 2, and 4. Subtype 1 (Multiple set 3) had aberrations in an increased number of pathways compared to other groups including signaling to RAS, FGFR1, VEGF and ERBB4. Subtype 2 had distinctive pathway aberration related to DNA replication including S Phase, DNA strand elongation, and leading strand synthesis pathways. The adjusted p-values with overlapped genes for each pathway were summarized in Table S5. Both PCA and pathway analysis designated that subtype 1 (Multiple set 3) had a distinct pattern of expressional aberrations. Then, we inspected the loading components (genes) that accounted for the transcriptomic variations of the Multiple set 3 (Fig. 3C). The PC1 contributed to 46.51% of total variations, and separated SNU-3698 C from SNU-3698 A and SNU-3698B, which indicated that loading components consisting of PC1 were responsible to spatially varying expression patterns. On the other hands, the PC2 accounting for 22.97% of entire variations divided SNU-3705 from SNU-3730 and SNU-3716, which represented the temporal heterogeneity. The entire composition of loading components is summarized in Table S6. We further estimated pathways that were specifically altered in accordance with multiple sampling by using top 10% of PC2 loading components. This revealed several tumor-related pathways including HIF-1, ErbB and MAPK signaling pathways (Fig. 3D, Table S7). Finally, we calculated the normalized enrichment scores (NES) for each multiple set using gene set enrichment analysis (GSEA) in order to estimate overall temporal transcriptomic changes of MPE samples (Fig. 3E, Table S8). The result suggested that most hallmark pathways were altered in patient-specific manners. The Multiple sets 2 and 4 underwent relatively radical expressional changes compared to sets 1 and 3. Notably, MYC_TARGETS_V2 signaling was significantly shifted in all sets except for set 2, which partially validated genes consisting of the MYC pathway were involved in subclonal selection in MPE. Multiple set 2 exhibited more pathway aberrations compared to other sets. We confirmed that epithelial-mesenchymal transition (EMT) and hedgehog signaling pathways were significantly dysregulated in set 2 (Fig. 3F). Overall, we confirmed that our cell line models of multiple sets recapitulated heterogeneous features of breast cancer. While notable differences were observed between patients, these findings are descriptive and should be interpreted in the context of each patient’s unique clinical background, including treatment history and disease stage.

### Molecular diversity caused by tumor heterogeneity affects drug responses

We integrated the actively shifting molecular features of the MPE microenvironment with variable drug responses. Twenty-three cancer cell lines and three organoids were successfully screened using a clinically-relevant 25 compounds library in biological triplicates generating more than 2,000 drug interactions. The detailed information of drug library and screening results are summarized in Table S9 and Methods section.

**Fig. 4:**
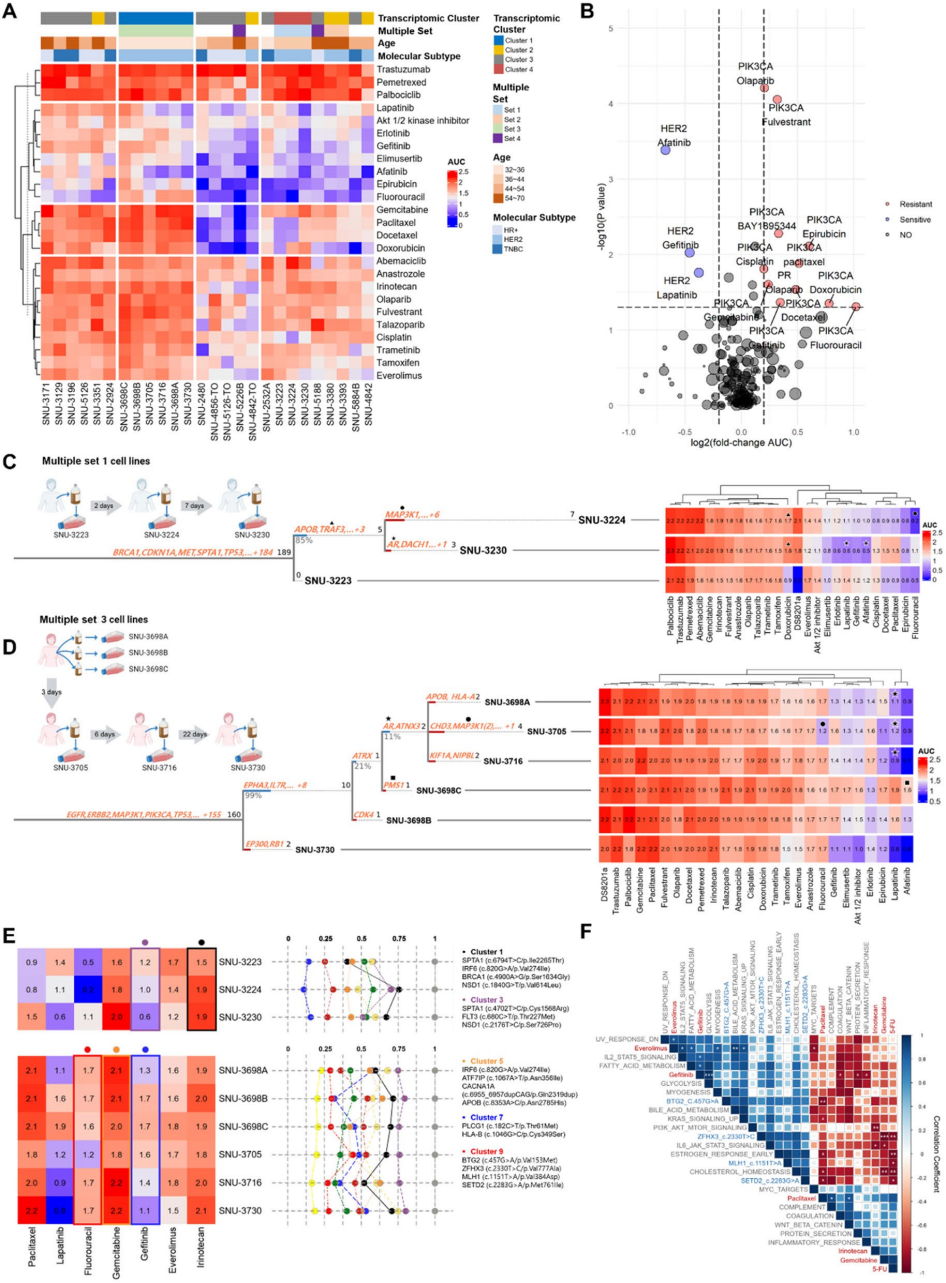
MPE-derived breast tumor *in-vitro* models reveal heterogeneous drug responses caused by molecular diversity. **A** See also Table S10 A. A heatmap of MPE-derived breast tumor *in-vitro* models exhibited heterogeneous distribution of 25 compounds according to their molecular characteristics. The names of compounds are provided on the right. The cell lines and drugs were k-means clustered based on the AUC values across the drug panel. Multiple factors that potentially contribute to the heterogeneous drug responses are indicated above the heatmap. The normality of each drug response was estimated with Shapiro–Wilk test. The *p*-values < 0.05 was considered as normally distributed data. **B** See also Table S10B, **C.** Gene-drug interaction analysis using Wilcoxon ranked sum test. Each dot indicated a pair of gene and drug. The size of circle is proportional to pair count. The absolute log fold change of AUC value > 0.2 and *p* < 0.05 were considered as significant. **C**, **D**. Multiple cell lines reveal heterogeneous drug responses caused by mutational ITH. Shifting mutational landscape during temporal evolution was associated to heterogeneous drug responses (marked with plane figures). The Treeomics statistical settings were as follows (sequencing error rate = 0.005, prior absent probability = 0.5, max absent VAF = 0.05, LOH frequency = 0, false discovery rate = 0.05, false-positive rate = 0.005, and absent classification minimum coverage = 100). **E**. Longitudinal tracking of mutational fraction revealed drug-associated variant clusters. Each cluster was highlighted with representative colors. Mutational clusters related to varying drug responses were marked with colored dot on the top of the drug heatmap. **F**. See also Table S11. Correlation plot shows the statistical association of molecular factors contributing to subclonal cell fractions including the VAFs of representative mutations and NES of specific pathways to certain drugs. The Pearson correlation coefficient (R) with *p*-values between the molecular factors and AUC of six drugs are represented. (Blue: positive correlation; Red: negative correlation). Significance codes: ‘***’ *p* < 0.001; ‘**’ *p* < 0.01; ‘*’ *p* < 0.05

Both cell lines and drugs were grouped into 4 transcriptomic clusters resulting in 16 cell lines-drugs associations (Fig. 4A). This classification revealed a group of drugs such as lapatinib, erlotinib, elimusertib, and afatinib showed different sensitivity in cell lines with different molecular features. It also demonstrated a few TNBC cell lines such as SNU-2924, SNU-3129, and SNU-3196 exhibited high level of resistance to most screened drugs, in parallel with prior findings [24]. To statistically evaluate the effect of such factors to each drug, we performed MANOVA. The transcriptomic cluster factor had a significant association with responses of drugs such as cisplatin (*p* < 0.001) and fulvestrant (*p* < 0.001) (Fig. 4A, Table S10 A). Since the cell lines within the Multiple sets represented the characteristics of a single patient, drugs that showed analogous responses within the Multiple sets were excluded from the analysis to avoid unwanted statistical weight. Then we further estimated mutation-drug interaction using Wilcox ranked sum test. We only counted the previously identified pathogenic mutations including *PIK3CA* and *TP53* (Fig. 4B, Table S10B and Table S10 C).

We traced the mutational evolution of temporal samples to explore how mutational diversity relates to varying drug responses. Phylogenetic analysis revealed that SNU-3223 evolved around mutations in the APOP and TRAF3 genes (Fig. 4C). Notably, both SNU-3224 and SNU-3230 harbored mutations in TRAF3, a gene associated with doxorubicin sensitivity [25]. Our Multiple Set models effectively recapitulated this specific gene–drug interaction (indicated with triangles). Similarly, only SNU-3230 carried a mutation in the AR gene, which is known to influence responses to lapatinib [26] and afatinib [27]. Consistent with prior findings, the AR mutation impacted the lapatinib response (indicated with stars in Fig. 4B). In addition to acquired mutations, changes in cellular fractions also contributed to substantial variability in drug responses (Fig. 4E). For instance, the variant allele frequencies (VAFs) of mutational clusters 5 and 9 showed strong correlations with the responses to gemcitabine and fluorouracil, respectively.

Finally, we linearly interlinked the variable drug responses with molecular diversities contributing to intratumoral heterogeneity of breast cancer. We manually screened drugs that exhibited significant variations in the multiple samples comprising irinotecan, everolimus, gefitinib, docetaxel, 5-FU, lapatinib, gemcitabine and paclitaxel. Those drugs were considered as potential targets that responded variably in accordance with previously described alteration of tumor cell fractions in MPE. Then, we performed Pearson correlation test in order to validate which molecular factors were linearly correlated to variable drug responses. This approach revealed six drugs (everolimus, gefitinib, paclitaxel, irinotecan, gemcitabine, and 5-FU) had significant correlation with determinant molecular factors (Fig. 4F, Table S11). For instance, the sensitivity of gemcitabine was inversely related to VAFs of *ZFHX3* (c.2330 TC/p.Val777 Ala). Previous study reported that *ZFHX3* is involved with proliferation of breast cancer cells by enhancing MYC and TBX3 transcription [28], which was well recapitulated in our data. Also, our result demonstrated that the response of gefitinib was directly connected to enrichment scores of glycolysis pathway. Prior research reported that GPR119 agonists such as MBX-2982 caused a metabolic shift to enhanced glycolysis accompanied by reduced mitochondrial oxidative phosphorylation, which resulted in better gefitinib responsiveness [29].

## Discussion

Tumor recurrence and metastasis following therapy are one of the most challenging difficulties in treatment of breast cancer, yet the changing clonal composition of breast tumor during relapse and metastasis remains uncertain [30]. Especially, breast cancer patients with MPE have a poor prognosis since tumor cells in MPE cause unpredictable resistance to chemotherapy and potentially invade into pleural membrane and adjacent organs [8, 9]. In order to access molecular heterogeneity within MPE, we established cell line models from multiply collected MPE samples with a time interval of few days and weeks, which were subjected to genomic, transcriptomic and drug sensitivity analysis. The observed heterogeneous drug responses and clonal evolution in our MPE-derived cell lines align with reports of anoikis resistance, EMT activation, and immune evasion as key drivers of therapeutic failure in MPE-complicated malignancies. Additionally, the variability in response to taxanes and DNA-damaging agents across our models supports previous observations that MPE microenvironments select for chemoresistant subclones with altered signaling pathways and stem-like features [31]. Our results extend these findings by providing a multi-omics perspective on how clonal dynamics and transcriptomic shifts correlate with drug sensitivity, emphasizing the potential of MPE-derived models as a platform for personalized drug screening and resistance prediction.

The different culture methods had a little influence on the mutational and expression profiles as the molecular patterns between cell lines and PDX-derived organoids (PDXO) were highly analogous (Fig. S2 and Fig. 3A). Nevertheless, PDXOs were grouped adjacent to each other within the same cluster, suggesting that the culture method affected responses to certain drugs including taxanes. For instance, the cell line, SNU-4842 exhibited moderate response to taxanes including paclitaxel and docetaxel, whereas the corresponding organoid, SNU-4842-TO was highly sensitive to taxanes. This implies that there is an intrinsic limitation when comparing drug screening results from 2 and 3D cultures. The patient for SNU-4842 received doxorubicin and cyclophosphamide before sampling of the pleural effusion (Table S1) and progressed with metastatic disease and received docetaxel with trastuzumab plus pertuzumab showing partial response. Between the two model systems, our results indicate that MPE-derived 2D cell cultures more faithfully reflected the clinical drug responses observed in patients, as exemplified by the SNU-2480 cell line, which demonstrated strong cisplatin resistance consistent with the patient’s treatment history. In contrast, while PDXO retained key molecular characteristics, their drug response profiles—particularly to agents such as taxanes—were sometimes more variable, possibly due to selection pressures during in vivo PDX expansion. These findings suggest that MPE-derived 2D cultures may serve as more immediate and reliable models for assessing patient-specific drug sensitivity, particularly in the context of cytotoxic chemotherapy.

Patients enrolled in this study received various palliative chemotherapies and pleural interventions before the establishment of the cell lines. Most of them received more than 1 or 2 lines of chemotherapies before sampling, which partially explains the drug screening results (Table S1). For instance, the patient for SNU-2480 was diagnosed with TNBC and was treated with neoadjuvant anthracycline and taxane containing therapy, but progressed with metastatic disease and received cisplatin containing regimen and capecitabine and the tumor progressed after several months of stable disease. In analogous with clinical data, SNU-2480 cell line exhibited strong resistance to Cisplatin (Fig. 4A). This re-confirms the value of the MPE-derived breast tumor cell line in predicting drug responses.

Multiply collected tumor cells from the same patients were mostly positioned within the same cluster in the drug heatmap, except for Multiple Set 4, which consisted of two cell lines: SNU-5188 collected before treatment with letrozole and palbociclib, and SNU-5226B collected after 2 weeks of treatment with letrozole and palbociclib (Fig. 4A). Although no heterogeneous cell population with respective of mutational VAFs was detected in the multiple set 4, the transcriptomic enrichment scores of multiple oncogenic pathways including DNA_REPAIR, E2 F_TARGETS, and MYC_TARGETS_V2 were decreased in SNU-5226B compared to SNU-5188 (Fig. 3E), which suggested that the treatment of palbociclib may have altered the tumor cell fractions within MPE in terms of the transcriptomic patterns. Indeed, prior clinical trials indicated that the E2F transcriptional pathway is associated with response to CDK4/6 inhibitors such as palbociclib [32].

While this study provides comprehensive genomic, transcriptomic, and drug sensitivity profiles of MPE-derived breast cancer models, we recognize the importance of further molecular characterization to enhance the translational relevance of these models. In future work, we aim to integrate quantitative proteomic analyses to complement the transcriptomic data and to elucidate functional protein-level alterations associated with drug responses. Additionally, the development of in vivo models incorporating immune components is being considered to explore immune-related signatures and their impact on therapeutic sensitivity. These efforts will contribute to establishing a multi-omics framework for non-invasively obtained MPE-derived tumor cells and facilitate the identification of personalized treatment strategies that are more closely aligned with clinical practice.

Overall, we have successfully established preclinical in vitro models using pleural effusion and demonstrated that the clonal fractions of breast tumors within MPE represent intratumoral heterogeneity. While clonal dynamics within individual patients highlight the adaptability of breast tumor cells in MPE, given the variability in clinical factors such as treatment regimens and tumor stage, differences observed across patients should not be generalized. Further studies involving larger cohorts are needed to elucidate whether common patterns of tumor evolution in MPE exist across diverse clinical contexts. Both VAFs of certain driver mutations and transcriptomic analysis identified potential targets that were statistically associated with heterogeneous drug responses. Our data suggest that multi-omics analysis on MPE-derived breast tumor cell lines represent a noninvasive and viable biological resource to provide molecular clue for determining chemotherapy regimen, taking both genetic and transcriptomic features into consideration. Our work emphasizes the importance of comprehensively characterizing the molecular features of MPE-complicated tumor cells in order to overcome potential drug resistance and metastasis associated with MPE.

## Supplementary Information

Additional file 1.

Additional file 2. Fig. S1. Mycoplasma test indicates all cell lines and organoids were also confirmed to be free of mycoplasma contamination.

Additional file 3. Fig. S2A. See also Table S3A and 3B. Genomic profiles of MPE-derived *in-vitro* models of breast cancer. Multiple somatic mutations including point mutations in putative tumor driver genes were identified. Each specific type of alteration is marked with representative colors. The known pathogenicity of selected mutations referred from ClinVar database is indicated by a plain figure on the waterfall plot. 2B. Graphical expression of clonal composition of SNU-3698A, SNU-3698B and SNU-3698C. 2C. Graphical expression of clonal composition of SNU-3705, SNU-3716 and SNU-3730.

Additional file 4. Table S1. Related to Fig. 4A and 4B. Detailed Clinical information of 15 enrolled breast cancer patients.

Additional file 5. Table S2. DNA finger printing analysis of 27 breast cancer *in-vitro* models.

Additional file 6. Table S3A. Related to Fig. S2. Representative mutations of 27 Breast Cancer Models. 3B. Related to Fig. S2. Confirmed pathogenicity using ClinVar database.

Additional file 7. Table S4A. Related to Fig. 2A. Cluster identification of multiple set 1. 4B. Related to Fig. 2B. Cluster identification of multiple set 2. 4C. Related to Fig .2C. Cluster identification of multiple set 3.

Additional file 8. Table S5. Related to Fig. 3B. Pathway analysis according to transcriptomic subtypes.

Additional file 9. Table S6. Related to Fig. 3C. Total loading components of multiple set 3.

Additional file 10. Table S7. Related to Fig. 3D. Clustered pathway analysis separates multiple pathways into biological groups.

Additional file 11. Table S8. Related to Fig. 3E. Enrichment scores with standard deviation for multiple sets.

Additional file 12. Table S9. Related to Fig. 4A. 25 clinically-relevant antitumor reagents with AUC values for each cell line.

Additional file 13. Table S10. Related to Fig. 4A. A. Multivariate Analysis of Variance with Transcriptomic Clusters with Shapiro-Wilk Normality Test. B. Wilcox ranked sum test with Mutation Status of PIK3CA. C. Wilcox ranked sum test with Mutation Status of TP53.

Additional file 14. Table S11. Related to Fig. 4B. Pearson correlation scores of drug responses to molecular factors.

## Data Availability

All data used in this work can be acquired from the corresponding author upon reasonable request.

## References

[1] Sung H, Ferlay J, Siegel RL, Laversanne M, Soerjomataram I, Jemal A, Bray F. Global Cancer Statistics 2020: GLOBOCAN Estimates of Incidence and Mortality Worldwide for 36 Cancers in 185 Countries. CA Cancer J Clin. 2021;71(3):209–49. 33538338 10.3322/caac.21660

[2] Bray F, Ferlay J, Soerjomataram I, Siegel RL, Torre LA, Jemal A. Global cancer statistics 2018: GLOBOCAN estimates of incidence and mortality worldwide for 36 cancers in 185 countries. CA Cancer J Clin. 2018;68(6):394–424. 30207593 10.3322/caac.21492

[3] Yates LR, Gerstung M, Knappskog S, Desmedt C, Gundem G, Van Loo P, Aas T, Alexandrov LB, Larsimont D, Davies H, et al. Subclonal diversification of primary breast cancer revealed by multiregion sequencing. Nat Med. 2015;21(7):751–9. 26099045 10.1038/nm.3886 PMC4500826

[4] Guo L, Kong D, Liu J, Zhan L, Luo L, Zheng W, Zheng Q, Chen C, Sun S. Breast cancer heterogeneity and its implication in personalized precision therapy. Exp Hematol Oncol. 2023;12(1):3. 36624542 10.1186/s40164-022-00363-1 PMC9830930

[5] Martelotto LG, Ng CK, Piscuoglio S, Weigelt B, Reis-Filho JS. Breast cancer intra-tumor heterogeneity. Breast cancer research : BCR. 2014;16(3):210. 25928070 10.1186/bcr3658 PMC4053234

[6] Esparza-López J, Escobar-Arriaga E, Soto-Germes S, Ibarra-Sánchez MJ. Breast Cancer Intra-Tumor Heterogeneity: One Tumor, Different Entities. Revista de investigacion clinica; organo del Hospital de Enfermedades de la Nutricion 2017, 69(2):66–76. 10.24875/ric.17002177 28453505

[7] Li A, Keck JM, Parmar S, Patterson J, Labrie M, Creason AL, Johnson BE, Downey M, Thomas G, Beadling C, et al. Characterizing advanced breast cancer heterogeneity and treatment resistance through serial biopsies and comprehensive analytics. NPJ precision oncology. 2021;5(1):28. 33772089 10.1038/s41698-021-00165-4 PMC7997873

[8] Guo Z, Xie Z, Shi H, Du W, Peng L, Han W, Duan F, Zhang X, Chen M, Duan J, et al. Malignant pleural effusion supernatant is an alternative liquid biopsy specimen for comprehensive mutational profiling. Thorac Cancer. 2019;10(4):823–31. 30779318 10.1111/1759-7714.13006 PMC6449231

[9] Zamboni MM, da Silva CT, Jr., Baretta R, Cunha ET, Cardoso GP,. Important prognostic factors for survival in patients with malignant pleural effusion. BMC Pulm Med. 2015;15:29. 25887349 10.1186/s12890-015-0025-z PMC4379612

[10] Paoli P, Giannoni E, Chiarugi P. Anoikis molecular pathways and its role in cancer progression. Biochem Biophys Acta. 2013;1833(12):3481–98. 23830918 10.1016/j.bbamcr.2013.06.026

[11] Saha T, Lukong KE. Breast Cancer Stem-Like Cells in Drug Resistance: A Review of Mechanisms and Novel Therapeutic Strategies to Overcome Drug Resistance. Front Oncol. 2022;12:856974. 35392236 10.3389/fonc.2022.856974 PMC8979779

[12] Dai Y, Zhang X, Ou Y, Zou L, Zhang D, Yang Q, Qin Y, Du X, Li W, Yuan Z, et al. Anoikis resistance–protagonists of breast cancer cells survive and metastasize after ECM detachment. Cell Commun Signal. 2023;21(1):190. 37537585 10.1186/s12964-023-01183-4 PMC10399053

[13] Seo HY, Kim SC, Roh WL, Shin YK, Kim S, Kim DW, Kim TM, Ku JL. Culture and multiomic analysis of lung cancer patient-derived pleural effusions revealed distinct druggable molecular types. Sci Rep. 2022;12(1):6345. 35428753 10.1038/s41598-022-10318-5 PMC9012760

[14] Cailleau R, Young R, Olivé M, Reeves WJ Jr. Breast tumor cell lines from pleural effusions. J Natl Cancer Inst. 1974;53(3):661–74. 4412247 10.1093/jnci/53.3.661 PMC7364228

[15] Tiran V, Stanzer S, Heitzer E, Meilinger M, Rossmann C, Lax S, Tsybrovskyy O, Dandachi N, Balic M. Genetic profiling of putative breast cancer stem cells from malignant pleural effusions. PLoS ONE. 2017;12(4):e0175223. 28423035 10.1371/journal.pone.0175223 PMC5396869

[16] Agalioti T, Giannou AD, Krontira AC, Kanellakis NI, Kati D, Vreka M, Pepe M, Spella M, Lilis I, Zazara DE, et al. Mutant KRAS promotes malignant pleural effusion formation. Nat Commun. 2017;8:15205. 28508873 10.1038/ncomms15205 PMC5440809

[17] Li H, Durbin R. Fast and accurate short read alignment with Burrows-Wheeler transform. Bioinformatics (Oxford, England). 2009;25(14):1754–60. 19451168 10.1093/bioinformatics/btp324 PMC2705234

[18] McKenna A, Hanna M, Banks E, Sivachenko A, Cibulskis K, Kernytsky A, Garimella K, Altshuler D, Gabriel S, Daly M, et al. The Genome Analysis Toolkit: a MapReduce framework for analyzing next-generation DNA sequencing data. Genome Res. 2010;20(9):1297–303. 20644199 10.1101/gr.107524.110 PMC2928508

[19] Bolger AM, Lohse M, Usadel B. Trimmomatic: a flexible trimmer for Illumina sequence data. Bioinformatics (Oxford, England). 2014;30(15):2114–20. 24695404 10.1093/bioinformatics/btu170 PMC4103590

[20] Kim D, Langmead B, Salzberg SL. HISAT: a fast spliced aligner with low memory requirements. Nat Methods. 2015;12(4):357–60. 25751142 10.1038/nmeth.3317 PMC4655817

[21] Pertea M, Pertea GM, Antonescu CM, Chang TC, Mendell JT, Salzberg SL. StringTie enables improved reconstruction of a transcriptome from RNA-seq reads. Nat Biotechnol. 2015;33(3):290–5. 25690850 10.1038/nbt.3122 PMC4643835

[22] Sondka Z, Bamford S, Cole CG, Ward SA, Dunham I, Forbes SA. The COSMIC Cancer Gene Census: describing genetic dysfunction across all human cancers. Nat Rev Cancer. 2018;18(11):696–705. 30293088 10.1038/s41568-018-0060-1 PMC6450507

[23] Landrum MJ, Lee JM, Benson M, Brown GR, Chao C, Chitipiralla S, Gu B, Hart J, Hoffman D, Jang W, et al. ClinVar: improving access to variant interpretations and supporting evidence. Nucleic Acids Res. 2018;46(D1):D1062-d1067. 29165669 10.1093/nar/gkx1153 PMC5753237

[24] Marra A, Trapani D, Viale G, Criscitiello C, Curigliano G. Practical classification of triple-negative breast cancer: intratumoral heterogeneity, mechanisms of drug resistance, and novel therapies. NPJ breast cancer. 2020;6:54. 33088912 10.1038/s41523-020-00197-2 PMC7568552

[25] Moore CR, Edwards SK, Xie P. Targeting TRAF3 Downstream Signaling Pathways in B cell Neoplasms. Journal of cancer science & therapy. 2015;7(2):67–74. 25960828 10.4172/1948-5956.1000327 PMC4422099

[26] Wahdan-Alaswad R, Liu B, Thor AD. Targeted lapatinib anti-HER2/ErbB2 therapy resistance in breast cancer: opportunities to overcome a difficult problem. Cancer drug resistance (Alhambra, Calif). 2020;3(2):179–98. 35582612 10.20517/cdr.2019.92 PMC9090587

[27] Schweizer MT, Yu EY. Persistent androgen receptor addiction in castration-resistant prostate cancer. J Hematol Oncol. 2015;8:128. 26566796 10.1186/s13045-015-0225-2 PMC4644296

[28] Dong G, Ma G, Wu R, Liu J, Liu M, Gao A, Li X, A J, Liu X, Zhang Z et al. ZFHX3 Promotes the Proliferation and Tumor Growth of ER-Positive Breast Cancer Cells Likely by Enhancing Stem-Like Features and MYC and TBX3 Transcription. Cancers 2020, 12(11):3415. 10.3390/cancers12113415 PMC7698617 33217982

[29] Im JH, Kang KW, Kim SY, Kim YG, An YJ, Park S, Jeong BH, Choi SY, Lee JS, Kang KW. GPR119 agonist enhances gefitinib responsiveness through lactate-mediated inhibition of autophagy. Journal of experimental & clinical cancer research : CR. 2018;37(1):295. 30497501 10.1186/s13046-018-0949-2 PMC6267899

[30] Walens A, Lin J, Damrauer JS, McKinney B, Lupo R, Newcomb R, Fox DB, Mabe NW, Gresham J, Sheng Z, et al. Adaptation and selection shape clonal evolution of tumors during residual disease and recurrence. Nat Commun. 2020;11(1):5017. 33024122 10.1038/s41467-020-18730-z PMC7539014

[31] Bhat GR, Sethi I, Sadida HQ, Rah B, Mir R, Algehainy N, Albalawi IA, Masoodi T, Subbaraj GK, Jamal F, et al. Cancer cell plasticity: from cellular, molecular, and genetic mechanisms to tumor heterogeneity and drug resistance. Cancer Metastasis Rev. 2024;43(1):197–228. 38329598 10.1007/s10555-024-10172-z PMC11016008

[32] Park YH, Im SA, Park K, Wen J, Lee KH, Choi YL, Lee WC, Min A, Bonato V, Park S, et al. Longitudinal multi-omics study of palbociclib resistance in HR-positive/HER2-negative metastatic breast cancer. Genome Med. 2023;15(1):55. 37475004 10.1186/s13073-023-01201-7 PMC10360358

