# Supplementary material for: Establishment and characterization of 24 breast cancer cell lines and 3 breast cancer organoids reveals molecular heterogeneity and drug response variability in malignant pleural effusion-derived models
Source: Breast Cancer Res. 2025 May 1;27:66. doi: 10.1186/s13058-025-02032-7 (PMC12044882; doi:10.1186/s13058-025-02032-7)
Supplement: Supplementary file 1 — Additional file 1. [file 13058_2025_2032_MOESM1_ESM.pdf]

2020.04.01 수

㊉ Breast cancer Myco test (Takara Kit)

P.C N.C 4856 2480 2532A 2924 3129 3171 3196 3223 3224 3230 3351 3380 3393

1500
1000
500
300
200
100

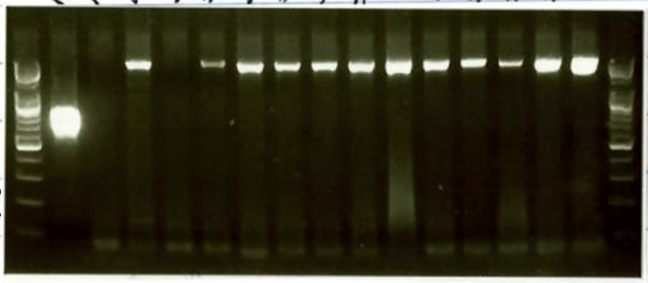

P.C N.C 3698A 3698B 3698C 3705 3716 3730 4842 5126 5188 5226B 5884B 4842-TO 4856-TO 5126-TO

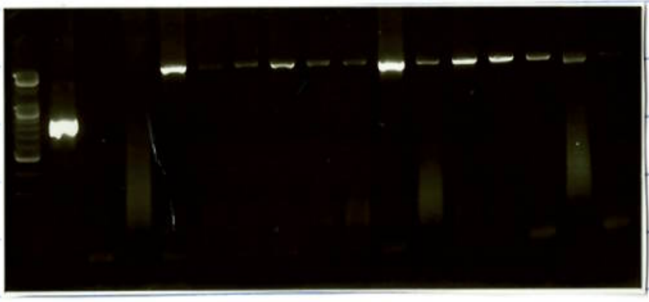

Myco band: 300-700 kDa

이렇게 loading됨

3380 5226B 5884B 4842-TO 4856-TO 5126-TO

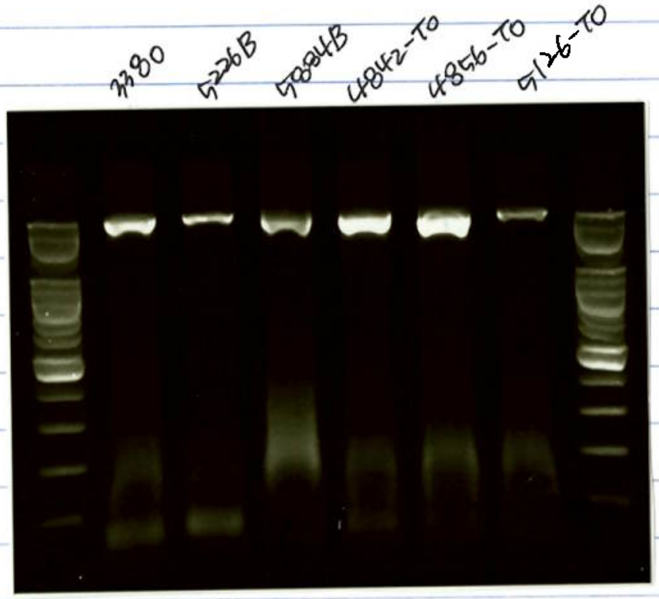

1500
1000
500
300
200
100

→ 위에서 쓴 DNA보다 더 later passage에 prep한 DNA들!

→ 모두 Myco 미검출! ✿
