# Supplementary figures and images for: Establishment and characterization of 24 breast cancer cell lines and 3 breast cancer organoids reveals molecular heterogeneity and drug response variability in malignant pleural effusion-derived models
Source: Breast Cancer Res. 2025 May 1;27:66. doi: 10.1186/s13058-025-02032-7 (PMC12044882; doi:10.1186/s13058-025-02032-7)

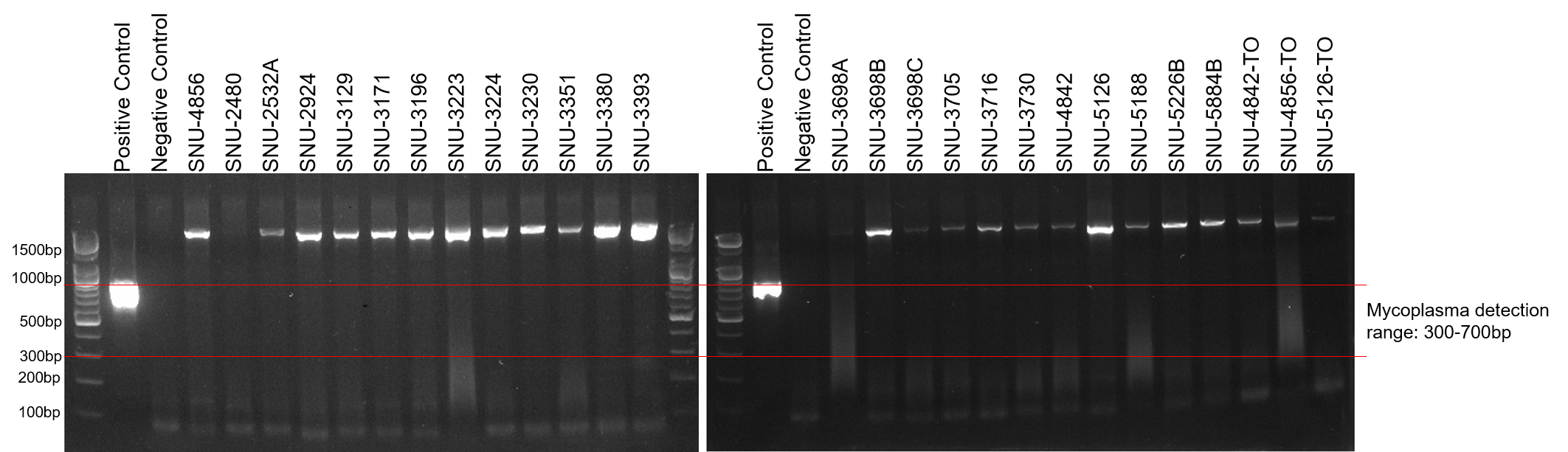

Supplement: Supplementary file 2 — Additional file 2. Fig. S1. Mycoplasma test indicates all cell lines and organoids were also confirmed to be free of mycoplasma contamination. [file 13058_2025_2032_MOESM2_ESM.tif]

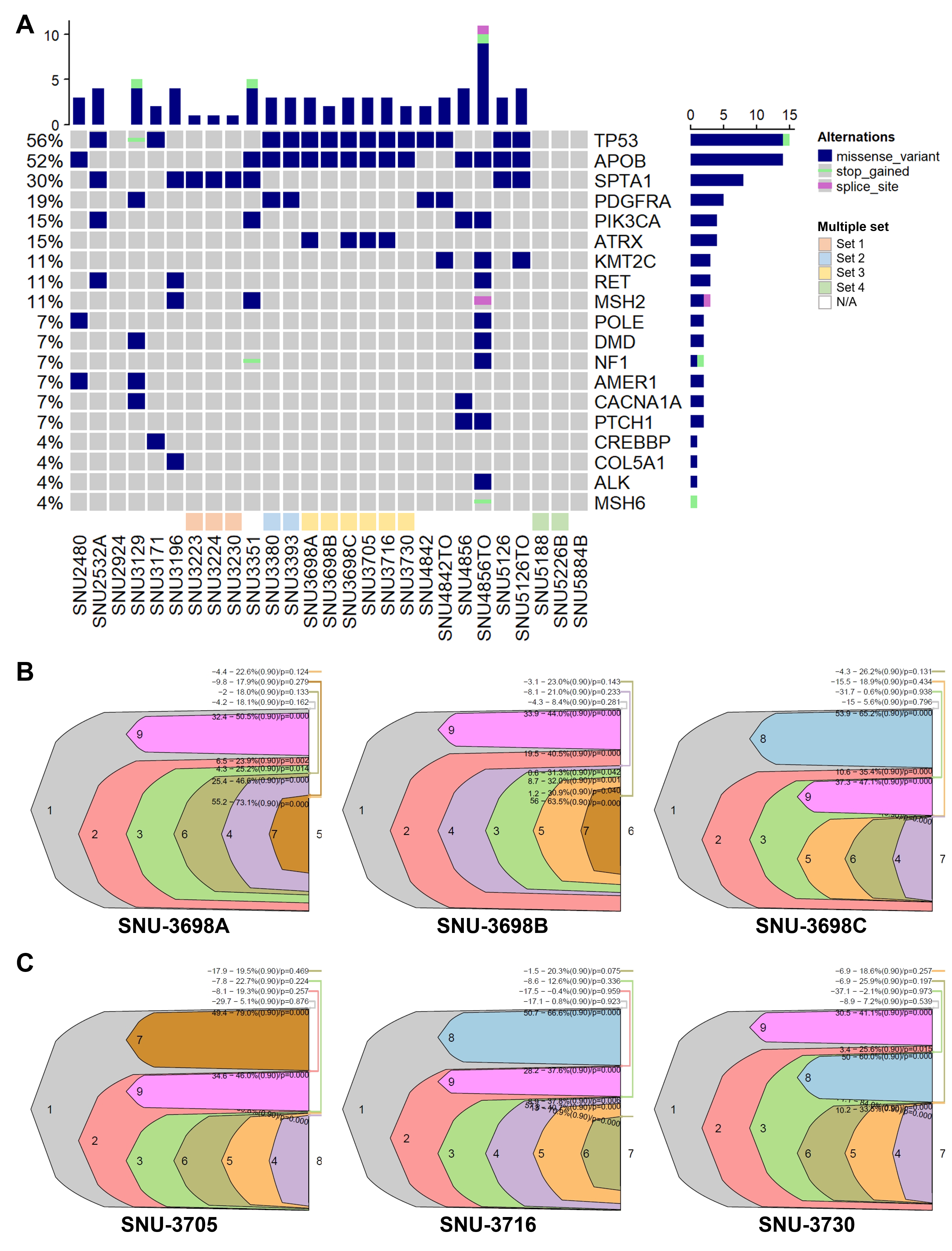

Supplement: Supplementary file 3 — Additional file 3. Fig. S2A. See also Table S3A and 3B. Genomic profiles of MPE-derived in-vitro models of breast cancer. Multiple somatic mutations including point mutations in putative tumor driver genes were identified. Each specific type of alteration is marked with representative colors. The known pathogenicity of selected mutations referred from ClinVar database is indicated by a plain figure on the waterfall plot. 2B. Graphical expression of clonal composition of SNU-3698A, SNU-3698B and SNU-3698C. 2C. Graphical expression of clonal composition of SNU-3705, SNU-3716 and SNU-3730. [file 13058_2025_2032_MOESM3_ESM.tif]
